# Exercise Intolerance in Pulmonary Arterial Hypertension

**DOI:** 10.1155/2012/359204

**Published:** 2012-06-10

**Authors:** Robin M. Fowler, Kevin R. Gain, Eli Gabbay

**Affiliations:** ^1^Advanced Lung Disease Program, Royal Perth Hospital, Level 3 Ainslie House, Murray Street, Western Australia 6000, GPO Box 2213, Perth, WA 6847, Australia; ^2^School of Physiotherapy and Curtin Health Innovation Research Institute, Curtin University, GPO Box U1987, Perth, WA 6845, Australia; ^3^Lung Institute of Western Australia (LIWA), Centre for Asthma, Allergy and Respiratory Research, University of Western Australia, Perth, WA 6009, Australia; ^4^Respiratory Medicine Department, Royal Perth Hospital, GPO Box 2213, Perth, WA 6847, Australia; ^5^School of Medicine and Pharmacology, University of Western Australia, Perth, WA 6009, Australia; ^6^School of Medicine, The University of Notre Dame, Perth, WA 6959, Australia; ^7^Heart & Lung Transplant Foundation of Western Australia, Perth, WA 6916, Australia

## Abstract

Pulmonary arterial hypertension (PAH) is associated with symptoms of dyspnea and fatigue, which contribute to exercise limitation. The origins and significance of dyspnea and fatigue in PAH are not completely understood. This has created uncertainly among healthcare professionals regarding acceptable levels of these symptoms, on exertion, for patients with PAH. Dysfunction of the right ventricle (RV) contributes to functional limitation and mortality in PAH; however, the role of the RV in eliciting dyspnea and fatigue has not been thoroughly examined. This paper explores the contribution of the RV and systemic and peripheral abnormalities to exercise limitation and symptoms in PAH. Further, it explores the relationship between exercise abnormalities and symptoms, the utility of the cardiopulmonary exercise test in identifying RV dysfunction, and offers suggestions for further research.

## 1. Introduction

Pulmonary arterial hypertension is a condition defined by primary abnormalities in the precapillary pulmonary arteries and arterioles. It forms group 1 of the World Health Organization classification of pulmonary hypertension (PH) [1]. This classification system identifies PAH as a specific entity, with a characteristic pathophysiology, clinical presentation, and response to therapy that helps separate it from other forms of pulmonary hypertension.

The most commonly reported symptoms on presentation in individuals with PAH are dyspnea and fatigue. These symptoms limit physical function, and, by the time of diagnosis, most individuals have marked functional limitation and are in the New York Heart Association (NYHA) Functional class III or IV [2]. The New York Heart Association reflects disease severity and prognosis, and disease progression is associated with worsening symptoms and functional capacity [1]. Recent development of pharmaceutical therapies, which address the specific pulmonary vascular abnormalities associated with PAH, has resulted in improved hemodynamics, exercise capacity [3, 4], and prognosis [3] for individuals with PAH. However, despite therapy, many individuals continue to have exertional symptoms, functional limitation and impaired quality of life (QoL) [5].

Exercise training has well-established safety and efficacy for improving exercise capacity and QoL in chronic obstructive pulmonary disease (COPD) [6] and left heart failure (LHF) [7]. Although, historically, physical activity and exercise training were discouraged for individuals with PAH, interest has recently developed in the role of exercise training for individuals with PAH who have persistent functional impairments, despite pharmaceutical therapy. Evidence from several small studies suggests that well-designed exercise training programs improve exercise capacity and QoL, without major adverse events or clinical deterioration, in individuals who are stable on PAH-specific pharmaceutical therapy [8–12]. These studies reporting exercise training have utilized moderate-intensity exercise.

In a study using a monocrotaline rat model of PAH, which investigated moderate intensity aerobic training [13], RV myocardial capillary density increased and exercise capacity improved following exercise training in rats with stable PAH. However, in rats in which progressive PAH had been induced with a higher dose of monocrotaline, signs of RV inflammation and poorer survival occurred following exercise training, in comparison with sedentary rats and rats with stable PAH which had undergone exercise training [13].

The paucity of literature reporting exercise training in PAH has resulted in uncertainty among healthcare professionals regarding appropriate levels of physical exertion for individuals with PAH, and which patients are suitable for exercise rehabilitation [14]. Furthermore, there is little in the literature regarding the causes and significance of dyspnea and fatigue associated with PAH. Consequently, healthcare professionals demonstrate inconsistency with respect to recommendations for appropriate levels of dyspnea and fatigue during the performance of daily activities in this population [14]. In light of the current interest in exercise training in PAH, it is timely that consideration be given to the hemodynamic consequences and origins and significance of the symptoms associated with physical exertion in PAH. This paper discusses the literature around exercise physiology in PAH, the likely impact of RV dysfunction and systemic and peripheral abnormalities on dyspnea, fatigue, and exercise limitation.

## 2. Central Hemodynamics in PAH

A fundamental endothelial abnormality is thought to play a key role in the pathogenesis and functional abnormalities associated with PAH. Imbalance in the production of pulmonary vasodilators and vasoconstrictors, abnormal proliferation of cells in the walls of the small pulmonary arteries and arterioles, and intra-luminal thrombus result in a marked reduction in the vasodilatory capacity, distensibility, and patency of the pulmonary circulation [15, 16]. The clinical outcome is a rise in pulmonary vascular resistance (PVR), pulmonary artery pressure (PAP), and RV afterload [17].

In a normal heart, the RV response to a sustained increase in afterload is adaptive myocardial hypertrophy. In PAH, with progressive vascular changes leading to an unrelenting increase in PVR, there is a transition from RV wall hypertrophy to RV dilatation [18]. The capacity for hypertrophic adaptation varies among individuals [19], and it has been proposed that the development of right heart failure in PAH is not only related to elevated RV afterload but also to intrinsic abnormalities of the RV wall [20] and may be related to myocardial inflammation [13]. Altered gene expression is thought to contribute to the development of RV dysfunction in some individuals [21]. In scleroderma, RV function can be further compromised by intrinsic abnormalities of the myocardium, which may be secondary to chronic inflammation [22]. However, the predominant cause of RV failure in PAH is believed to be RV ischemia due to imbalance between oxygen supply and demand associated with hypertrophy, increased RV workload and increased metabolic demand [23], without a concomitant increase in capillarization [13, 18, 20, 24] and blood supply [25].

Initially, dilatation of the right atrium and RV in PAH results in a compensatory increase in preload and maintenance of stroke volume (SV), but as contractile dysfunction worsens, diastolic dysfunction develops, filling pressures rise, and RV output falls [26]. The resultant decrease in left ventricular (LV) preload [27] and pressure-related movement of the interventricular septum to the left and LV compression [28], lead to a fall in LV output and systemic oxygen delivery [29, 30].

## 3. Exercise Abnormalities

Impairment in the distensibility and vasodilatory capacity and reduction in the size of the pulmonary vascular bed mean that an increase in pulmonary blood flow with exercise can only be achieved with a marked rise in PAP [31] and RV afterload [17]. Reduced RV contractility results in a reduced capacity for SV to augment cardiac output (CO) during exercise [30]. In addition to reduced SV, PAH is associated with chronotropic impairment [32], demonstrated by a failure to achieve a normal maximum heart rate at peak exercise [32–35]. Chronotropic impairment in PAH is related to downregulation of RV myocardial beta-adrenoreceptor activity [36] and reflects disease severity [32, 37]. The combined failure of SV and heart rate to increase normally during exercise results in an attenuated rise in CO and systemic blood pressure [38]. Prognosis in PAH is known to be closely associated with RV function [26] and the systemic blood pressure response during exercise [38]. Ultimately the RV fails to function adequately at rest, and, in the majority of cases, death occurs from RV failure [21].

## 4. The Influence of Right Ventricular Function on Exercise Capacity and Symptoms

There is increasing awareness that the primary cause of symptoms [39], functional impairment and mortality in PAH is RV dysfunction [23]. Along with being strongly associated with survival [40, 41], right atrial pressure has been identified as the hemodynamic measure that has the strongest (negative) correlation with exercise capacity in individuals with PAH [42]. Furthermore, indicators of RV function, SV and chronotropic response, are strong and independent factors in determining the six-minute walk distance (6MWD) [32]. Improvements in 6MWD are positively related to changes in SV, and chronotropic response [32] and cardiac index [17] and negatively related to changes in PVR and the Borg scale rating of dyspnea following PAH-specific therapy [32]. Treatments that improve hemodynamics by unloading the RV, and/or improving RV contractility, have also been shown to improve NYHA functional class [17].

Further insights into the role of the RV in the generation of symptoms and reduction in exercise capacity can be gained from studies in patients with left heart failure (LHF). Pulmonary hypertension, due to elevated pulmonary venous pressure, is commonly associated with LHF [43, 44]. While there is a poor correlation between exercise capacity and left ventricular function in LHF [45], RV function influences both exercise capacity and prognosis in this condition [46]. Resting PAP and PVR correlate inversely, and right ventricular ejection fraction correlates positively with peak oxygen consumption (VO_2_) [47, 48]. A high prevalence of PH has also been reported in chronic obstructive pulmonary disease (COPD) [49, 50] and pulmonary fibrosis [51, 52]. In these conditions, and in LHF, exercise capacity is lower and levels of dyspnea and fatigue are greater in individuals with pulmonary hypertension than those without [50, 52–55].

Recently, a study of individuals with normal hemodynamics at rest, but a persistent reduction in exercise capacity following successful pulmonary endarterectomy for chronic thromboembolic disease, was undertaken to investigate the cause of persistent exertional dyspnea and functional limitation [56]. This study identified elevated PVR and reduced pulmonary arterial compliance during exercise, and reduced exercise capacity in these individuals, in comparison with a control group. The combination of PVR and pulmonary arterial compliance reflects the hydraulic load imposed by the pulmonary circulation on the RV, and the findings of this study support the suggestion that elevated RV afterload negatively impacts on exercise capacity and contributes to exertional dyspnea [56].

The RV most likely contributes to the sensation of dyspnea via mechanoreceptors situated in the right atrium and RV. These receptors relay details of right atrial and RV pressure and volume and the amount of work performed by the RV [57, 58], via afferent sympathetic pathways, to the central nervous system. In PAH an increase in sympathetic activity [59] appears directly related to the degree of elevation of right atrial [60] or RV systolic pressure [61]. In animal models, sympathetic pathways have been implicated in mediating the association between RV work load and ventilatory response [62], with increased RV pressure, and stimulation of mechanoreceptors in the right atrium, directly resulting in increased ventilation [62, 63].

## 5. Other Abnormalities That Contribute to Reduced Exercise Capacity and Symptoms in PAH

### 5.1. Gas Exchange and Hypoxemia

Reduced diffusing capacity for carbon monoxide (DLCO) is a common finding in PAH [41, 64–67]. Reduced DLCO appears to be related primarily to impaired pulmonary membrane diffusing capacity and, to a lesser extent, reduced pulmonary capillary blood volume [66, 67]. Reduced DLCO has been shown to correlate with reduced exercise capacity and a higher functional class in PAH [68], likely reflecting disease severity. However, reduced DLCO also indicates a limited capacity for pulmonary gas exchange. In individuals with moderate to severe PAH, without a patent foramen ovale, a progressive fall in oxygen saturation occurs during exercise [35, 38]. It has been proposed that this results from reduced venous oxygen saturation secondary to reduced CO and tissue oxygen delivery [69]. At rest, mixed venous oxygen saturation has been shown to correlate with arterial oxygen tension (PaO_2_) [70, 71]. However, reduced oxygen uptake in the lung secondary to rapid red cell transit time, diffusion impairment [66], and ventilation/perfusion mismatch [70, 72] also contributes to hypoxemia.

Hypoxemia stimulates ventilation through central chemoreceptors in the medulla and peripheral chemoreceptors in the carotid and aortic bodies. However, central chemoreceptors are generally only stimulated when PaO_2_ is close to, or below, 50 mmHg [73]. There are conflicting data in the literature regarding a correlation between the ventilatory response (represented by the ventilatory equivalent for carbon dioxide [$\dot{\text{V}}$E/$\dot{\text{V}}$CO_2_]) during exercise and arterial oxygen tension (PaO_2_), in individuals with PAH. Although early studies identified no correlation between $\dot{\text{V}}$E/$\dot{\text{V}}$CO_2_ and PaO_2_ [74, 75], a recent study identified a correlation at rest and at the anaerobic threshold [71]. Both elevated $\dot{\text{V}}$E/$\dot{\text{V}}$CO_2_ and reduced PaO_2_ reflect disease severity in PAH [38, 71], and a direct link between the ventilatory response and hypoxemia in this condition has not been established. In LHF, hyperventilation during exercise occurs in the absence of hypoxemia [76]. Except in the presence of a patent foramen ovale or severe disease, the levels of hypoxemia in PAH are insufficient to stimulate hypoxia sensitive central chemoreceptors, and it is, therefore, unlikely that hypoxemia makes a significant contribution to hyperventilation in the majority of individuals with PAH.

Hypoxemia may, however, contribute to a sensation of dyspnea by predisposing the respiratory muscles to fatigue. In healthy individuals undergoing prolonged exercise, fatigue-induced changes in the contractile properties of the respiratory muscles contribute to a sensation of dyspnea through imbalance in inspiratory muscle effort relative to capacity [77]. The dyspnea associated with central nervous system's perception of inspiratory motor output, relative to capacity, is also influenced by a reduction in respiratory muscle strength [78]. Respiratory muscle weakness has been demonstrated in PAH [79, 80], and there is evidence of atrophy of type I and type II muscle fibres in the diaphragm of humans with PAH [81]. In the presence of hypoxemia, along with elevated ventilation, respiratory muscle weakness, and reduced CO, the respiratory muscles are predisposed to fatigue, which may contribute to the sensation of dyspnea during exercise in PAH.

### 5.2. Chemoreceptor Activation

It is likely that reduced oxygen delivery contributes to increased ventilation and dyspnea in PAH via activation of skeletal muscle chemoreceptors. Reduced muscle cell pH associated with anaerobic metabolism stimulates intra- and extra-cellular chemoreceptors within the muscle and, via the ergoreflex, results in increased ventilation [82, 83]. In LHF, in the longer term, reduced CO during exercise, and chronic muscle acidosis [84] result in increased ergoreflex sensitivity [85–87] and increased ventilation and dyspnea [45]. It has been proposed that peripheral chemoreceptor stimulation [59] and possibly increased ergoreflex sensitivity also contribute to increased ventilation and dyspnea in PAH, although there are no data to confirm this possibility, to date.

### 5.3. Systemic Endothelial Dysfunction

Tissue oxygen delivery and aerobic metabolism depend upon adequate systemic vascular function, along with CO and arterial oxygen content. Due to the influence of the systemic endothelium on vascular tone and blood flow, endothelial dysfunction is believed to negatively impact on oxygen delivery to the periphery in LHF [88–90]. Evidence of systemic endothelial dysfunction in PAH [91] suggests that reduced peripheral blood flow may also be a source of impaired oxygen delivery, muscle acidosis, and elevated ventilation, during exercise, in PAH.

### 5.4. Skeletal Muscle Myopathy

Recent studies have identified muscle fibre changes and skeletal muscle weakness in individuals with PAH [92, 93]. The muscle fibre changes include a lower portion of type I muscle fibres and an enzyme profile compatible with a relatively higher potential for anaerobic than aerobic energy metabolism [93]. The cause of skeletal muscle dysfunction in PAH is uncertain, although it is likely related to chronic muscle acidosis, increased sympathetic activity [59, 61], systemic inflammation [94, 95], and neurohormonal changes [18], similar to the causes of skeletal muscle dysfunction in LHF [96]. Similarities in muscle dysfunction in LHF, COPD, and PAH also suggest that skeletal muscle atrophy and alterations in muscle morphology in PAH may contribute to an elevated ventilatory drive, and dyspnea, as described in LHF and COPD [45, 97, 98]. The improvement in muscle morphology and exercise capacity following exercise training in PAH [10, 11] suggests that deconditioning also contributes to exercise limitation in PAH.

## 6. Ventilatory Response in PAH

Characteristic ventilatory abnormalities have been well defined in PAH. Hyperventilation at rest and on exercise, identified by an elevated $\dot{\text{V}}$E/$\dot{\text{V}}$CO_2_ and reduced arterial carbon dioxide tension (PaCO_2_), is a well-recognised feature of PAH [35, 38, 74, 75, 99]. The elevated $\dot{\text{V}}$E/$\dot{\text{V}}$CO_2_ in PAH describes a dissociation between carbon dioxide production, PaCO_2_, and minute ventilation. The altered relationship between $\dot{\text{V}}$E/$\dot{\text{V}}$CO_2_, PaCO_2_, and arterial pH described in PAH [74] suggests that elevated $\dot{\text{V}}$E/$\dot{\text{V}}$CO_2_ during submaximal exercise in PAH is not mediated by changes in arterial blood gases. Initial reports of an elevated $\dot{\text{V}}$E/$\dot{\text{V}}$CO_2_ suggested that increased ventilation in PAH was due to ventilatory inefficiency caused by obstruction of the small pulmonary vessels and subsequent ventilation/perfusion inequalities [74, 75, 99]. However, this is unlikely to be the predominant mechanism, as ventilation/perfusion studies in PAH do not demonstrate marked ventilation/perfusion mismatch, at rest or on exercise [70, 100]. Furthermore, in PAH it is well established that PaCO_2_ is reduced at rest and on exercise [40, 71]. If ventilatory inefficiency was the sole cause of an elevated $\dot{\text{V}}$E/$\dot{\text{V}}$CO_2_, PaCO_2_ would be normal. An increased ventilatory drive, rather than ventilatory inefficiency, is likely to be reflected in an elevated $\dot{\text{V}}$E/$\dot{\text{V}}$CO_2_ in the presence of a reduced PaCO_2_, as seen in PAH. This hypothesis warrants further investigation.

There is evidence that the elevated ventilatory response associated with PAH is related to central haemodynamic abnormalities. The $\dot{\text{V}}$E/$\dot{\text{V}}$CO_2_ at rest has been shown to correlate with PVR, and both $\dot{\text{V}}$E/$\dot{\text{V}}$CO_2_ and PVR decrease in response to treatment with an intravenous prostacyclin analogue [101]. The $\dot{\text{V}}$E/$\dot{\text{V}}$CO_2_ correlates with PAP [75]. Arterial carbon dioxide tension has been shown to correlate with cardiac index and changes in cardiac index associated with disease progression and increasing PVR are reflected by changes in both $\dot{\text{V}}$E/$\dot{\text{V}}$CO_2_ and PaCO_2_ [71]. The $\dot{\text{V}}$E/$\dot{\text{V}}$CO_2_ reflects disease severity and has been shown to correlate with NYHA functional class [35]. Furthermore, the $\dot{\text{V}}$E/$\dot{\text{V}}$CO_2_ [38], and PaCO_2_ are both prognostic markers in PAH [71].

In LHF, RV workload, indirectly determined by measurement of RV oxidative metabolism [102, 103] and PVR [53, 104], correlates with $\dot{\text{V}}$E/$\dot{\text{V}}$CO_2_. Furthermore, in this condition, a significant negative relationship exists between RV ejection fraction and $\dot{\text{V}}$E/$\dot{\text{V}}$CO_2_ [104]. Changes in exercise PVR following treatment with the phosphodiesterase inhibitor, sildenafil, also correlate significantly with changes in $\dot{\text{V}}$E/$\dot{\text{V}}$CO_2_ [105] although there is no correlation between left ventricular function at peak exercise and $\dot{\text{V}}$E/$\dot{\text{V}}$CO_2_ [104]. Furthermore, the increase in $\dot{\text{V}}$E/$\dot{\text{V}}$CO_2_ reflects the degree in elevation of PAP [106] supporting a relationship between RV work, ventilatory response, and symptoms in this condition.

A distinct pattern of change in end-tidal carbon dioxide tension (PetCO_2_) during exercise is evident in individuals with PAH. In severe PAH, PetCO_2_ is low at rest and falls progressively throughout an incremental exercise test [31, 107, 108], most likely reflecting a low and falling PaCO_2_ at rest and on exercise, respectively. During recovery PetCO_2_ rises, reflecting slowed gas exchange kinetics and delayed recovery [31]. In moderate PAH the rise in PetCO_2_ from rest to the anaerobic threshold (AT) is minimal, or absent, and in mild PAH the rise in PetCO_2_ from rest to the AT is attenuated [108]. This particular pattern of PetCO_2_ response distinguishes PAH from other conditions [107].

## 7. Evidence of RV Dysfunction on a Cardiopulmonary Exercise Test (CPET) in Individuals with PAH

In PAH, the incremental CPET consistently identifies reduced peak oxygen consumption and reduced VO_2_ at the AT [31, 35, 38, 99, 109], reduced oxygen (O_2_) pulse [35, 38, 110], and slowed VO_2_ kinetics [31]. The relationship between CO and oxygen consumption is very strong in healthy individuals, such that VO_2_ is considered a surrogate of CO and VO_2_/HR, or O_2_ pulse has been used as a surrogate of SV [111]. Reduced VO_2_ at peak exercise and AT, reduced O_2_ pulse, and slowed VO_2_ kinetics during and following exercise reflect RV dysfunction, reduced CO, and an oxygen deficit during exercise [31, 111]. Oxygen desaturation reflects reduced mixed venous oxygen saturation (along with reduced O_2_ uptake in the lungs), further reflecting reduced CO and inadequate O_2_ delivery [69]. The well-described elevation in $\dot{\text{V}}$E/$\dot{\text{V}}$CO_2_ [17, 31, 35, 75, 99, 101, 108, 109] and the relationship between $\dot{\text{V}}$E/$\dot{\text{V}}$CO_2_ and cardiac function described in PAH suggest that high values of $\dot{\text{V}}$E/$\dot{\text{V}}$CO_2_ reflect high levels of RV pressure and workload [75, 101]. Low and falling PetCO_2_ at rest and during exercise reflect low levels of PaCO_2_ [71] associated with a ventilatory drive that is disconnected from carbon dioxide production. Low PetCO_2_ is also suggestive of hyperventilation related to elevated RV pressure and workload.

## 8. Exercise Abnormalities and the Functional Consequences of Exercise-Induced PAH

Invasive evaluation of central hemodynamics during exercise identifies individuals who do not meet the diagnostic criteria for PAH but who have an elevated pulmonary artery pressure and reduced CO at peak exercise (exercise-induced PAH (EIPAH)) [112, 113]. These individuals demonstrate abnormalities during exercise which are characteristic of the changes seen in PAH, albeit of a milder severity [114]. In comparison to a healthy control group, individuals with EIPAH have reduced peak VO_2_, reduced VO_2_ at AT [112, 113], reduced O_2_ pulse (Fowler et al., unpublished data), and a tendency towards arterial desaturation [113]. Individuals with EIPAH also demonstrate elevated $\dot{\text{V}}$E/$\dot{\text{V}}$CO_2_, reduced PetCO_2_ at the AT, and an attenuated rise in PetCO_2_ from rest to the AT [113]. A higher proportion of these individuals terminate exercise because of dyspnea, compared with matched healthy controls (41% versus 5%, resp.) [113]. Furthermore, individuals with EIPAH are in NYHA functional class II or III and have reduced 6MWD [115] and QoL [113] and lower limb muscle strength compared with healthy individuals [116]. While it is uncertain whether EIPAH is a progressive pulmonary vasculopathy similar to PAH, it is apparent that exercise abnormalities identified during formal exercise testing reflect a similar mechanism of exercise limitation, signs consistent with impaired RV function during exercise, and possibly early systemic sequelae of a pulmonary vasculopathy (including muscle dysfunction), as described in PAH.

## 9. The Relationship between Ventilation and Dyspnoea

The relationship between ventilation and dyspnea is well established, from studies of healthy individuals during exercise and in individuals with disease. Afferent neural input relays details of ventilation from respiratory muscle spindles to the respiratory centre in the medulla [117]. Ventilation during rest and light exercise occurs with little or no awareness of breathing [118]. However, an increase in motor command to ventilatory muscles is perceived as a sensation of respiratory work/effort, or dyspnea [78], and the increase in ventilation required to perform moderate or intense exercise is accompanied by an increasing awareness of breathing to a point where breathlessness is described, even in healthy subjects [118]. An individual with PAH has a greater ventilatory demand and minute ventilation throughout submaximal exercise and registers an awareness of breathing during lower levels of exercise than a healthy individual [35]. This describes an association between elevated ventilation and dyspnea in PAH.

## 10. Factors That Contribute to Fatigue in PAH

A sensation of fatigue is commonly reported in LHF, COPD, and PAH and is described as the limiting factor during exercise testing in up to half of individuals with these conditions [35, 119]. In LHF, muscle fatigue and early termination of exercise have been shown to be directly associated with reduced CO and leg blood flow and increased arterial lactate concentrations [120]. Through these mechanisms, reduced CO is considered to influence the sensation of general fatigue in individuals with LHF. It has been proposed that slowed VO_2_ kinetics and oxygen deficit in individuals with PAH are associated with similar depletion of high-energy compounds in the muscle as in LHF [31].

A change in muscle fibre proportion, with a reduction in type I and an increase in type II muscle fibres [93], results in reduced aerobic capacity, early anaerobic metabolism, and an increased propensity for fatigue in the muscles in PAH. Similar changes in muscle morphology and function in LHF and COPD are believed to be important factors contributing to the sensation of fatigue during exercise and reduced exercise capacity, in these conditions [121]. The skeletal muscle abnormalities identified in PAH [93] are also likely to contribute to the sensation of fatigue associated with this condition.

## 11. Summary and Conclusions

An acute increase in PAP and RV workload, in association with reduced oxygen delivery during exercise, and the longer term systemic and peripheral sequelae of PAH contribute to increased ventilation during exercise in individuals with PAH. The sensation of dyspnea reflects elevated ventilation during exercise and represents a limited capacity for increasing CO to meet the elevated metabolic demands of physical activity. While longer-term sequelae of reduced CO and tissue oxygenation contribute to fatigue in PAH, in the short term, fatigue signifies inadequate tissue oxygen delivery related to an attenuated rise in CO during exercise.

The symptoms of dyspnea and fatigue associated with PAH reflect both acute and chronic RV dysfunction, influence functional class, and, indirectly, predict survival. The level of these symptoms on exertion is used by clinicians to grade disease severity and prognosis in individuals with PAH. Clinicians are encouraged to also use these symptoms to guide and monitor the response to physical activities and exercise training in individuals with PAH. Severe dyspnea and fatigue are likely to reflect high levels of RV work, which exceed RV capacity, and which potentially contribute to RV ischemia, inflammation and progressive RV failure in individuals in whom there is active disease progression.

A CPET identifies findings consistent with RV dysfunction during exercise in individuals with PAH. A CPET also identifies a pulmonary vasculopathy and impaired RV function during exercise in symptomatic individuals who do not meet the diagnostic criteria for PAH. The CPET is encouraged as a tool to identify the functional consequences of PAH, to stratify symptomatic individuals for invasive evaluation, and for longitudinal followup in individuals who do not have PAH on initial assessment but who are at increased risk for developing PAH.

The evidence from exercise training studies, to date, suggests that, at least in the short term, exercise training at moderate intensity is associated with improved exercise capacity, without adverse outcomes, in individuals who are stable on PAH-specific therapy. For individuals with PAH who intend to undertake an exercise training program, wherever possible, a prior CPET is encouraged. A CPET allows the opportunity to screen individuals for risks associated with exercise (e.g., an abnormal blood pressure or heart rate response) and allows accurate determination of exercise intensity. The exercise intensity employed during training should be prescribed according to the individuals' CPET results, including the maximum heart rate response (especially in light of chronotropic impairment in PAH) and symptomatic responses at submaximal and maximal exercise. Clinicians are strongly encouraged to utilize symptoms to monitor and guide exercise workload and physical activity levels. Increasing or severe fatigue and/or severe dyspnea during exercise suggest a high level of RV work, which may have a detrimental impact on RV function.

## 12. Future Research

While there are data which describe exercise limitation and provide insights into the likely origin and symptoms associated with PAH, further research is required to confirm and expand these findings. This research might include studies to clarify the role of central hemodynamics and the RV in the origin of symptoms and exercise limitation in this population. Invasive measures of RV function during exercise are feasible, can be performed without adverse events and offer insights into the hemodynamic responses associated with exercise. Evaluation of the role of the central ventilatory drive, chronic muscle acidosis, the ergoreflex, and muscle dysfunction (including the role of deconditioning) would also be of value.

Complementary studies exploring the mechanisms by which exercise training improves symptoms, exercise capacity, and QoL are also required. Further studies are needed to determine the optimal intensity for exercise training and the appropriate level of symptoms during physical activity for individuals with PAH. These studies should include randomised controlled trials directed at determining the longer-term outcomes of exercise training on central hemodynamics, RV function, disease progression, exercise capacity, and QoL. Trial endpoints might include measures of RV function (ideally using magnetic resonance imaging, invasive hemodynamics or echocardiography), the association between symptoms and RV function, biomarkers such as brain natriuretic peptide, QoL, longer-term changes in physical function and usual activity levels, peripheral endothelial function, muscle strength, endurance and morphology (according to the exercise modality studied), and the ventilatory response during submaximal and maximal exercise testing.

Previous work in animal models of PAH suggests that exercise training trials in animal models are feasible and useful. The findings suggest that studies of exercise training in animal models may allow exploration of histological consequences of training, and exploration of exercise intensities that are currently considered potentially unsafe in human studies. Further exploration of the utility of ventilatory response during exercise as a surrogate for RV function would also be of value in animal, and human, studies.
